# Vitamin D3 ameliorates nitrogen mustard‐induced cutaneous inflammation by inactivating the NLRP3 inflammasome through the SIRT3–SOD2–mtROS signaling pathway

**DOI:** 10.1002/ctm2.312

**Published:** 2021-02-14

**Authors:** Xunhu Dong, Ying He, Feng Ye, Yuanpeng Zhao, Jin Cheng, Jingsong Xiao, Wenpei Yu, Jiqing Zhao, Yan Sai, Guorong Dan, Mingliang Chen, Zhongmin Zou

**Affiliations:** ^1^ Department of Chemical Defense Medicine, School of Military Preventive Medicine Third Military Medical University (Army Medical University) Chongqing China; ^2^ Institute of Toxicology, School of Military Preventive Medicine Third Military Medical University (Army Medical University) Chongqing China; ^3^ Institute of Pathology and Southwest Cancer Centre, Southwest Hospital Third Military Medical University (Army Medical University) Chongqing China; ^4^ Department of Ultrasound Xinqiao Hospital Third Military Medical University (Army Medical University) Chongqing China

**Keywords:** cutaneous inflammation, nitrogen mustard, NLRP3 inflammasome, SIRT3, vitamin D3

## Abstract

Nitrogen mustard (NM) causes severe skin injury with an obvious inflammatory response, which is lack of effective and targeted therapies. Vitamin D3 (VD3) has excellent anti‐inflammatory properties and is considered as a potential candidate for the treatment of NM‐induced dermal toxicity; however, the underlying mechanisms are currently unclear. Cyclooxygenase‐2 (COX2; a widely used marker of skin inflammation) plays a key role in NM‐induced cutaneous inflammation. Herein, we initially confirmed that NM markedly promoted COX2 expression in vitro and in vivo. NM also increased NOD‐like receptor family pyrin domain containing 3 (NLRP3) expression, caspase‐1 activity, and interleukin‐1β (IL‐1β) release. Notably, treatment with a caspase‐1 inhibitor (zYVAD‐fmk), NLRP3 inhibitor (MCC950), and *NLRP3* or *caspase‐1* siRNA attenuated NM‐induced NLRP3 inflammasome activation, with subsequent suppression of COX2 expression and IL‐1β release in keratinocytes. Meanwhile, NM increased mitochondrial reactive oxygen species (mtROS) and decreased manganese superoxide dismutase 2 (SOD2) and sirtuin 3 (SIRT3) activities. Mito‐TEMPO (a mtROS scavenger) ameliorated NM‐caused NLRP3 inflammasome activation in keratinocytes. Moreover, VD3 improved SIRT3 and SOD2 activities, decreased mtROS contents, inactivated the NLRP3 inflammasome, and attenuated cutaneous inflammation induced by NM in vitro and in vivo. The beneficial activity of VD3 against NM‐triggered cutaneous inflammation was enhanced by the inhibitors of IL‐1, mtROS, NLRP3, caspase‐1, and *NLRP3* or *caspase‐1* siRNAs, which was abolished in SIRT3 inhibitor or *SIRT3* siRNA‐treated keratinocytes and skins from SIRT3^−/−^ mice. In conclusion, VD3 ameliorated NM‐induced cutaneous inflammation by inactivating the NLRP3 inflammasome, which was partially mediated through the SIRT3–SOD2–mtROS signaling pathway.

## Graphical abstract

Vitamin D3 (VD3) has excellent anti‐inflammatory properties and is considered as a potential candidate for the treatment of nitrogen mustard (NM)‐induced dermal toxicity; however, the underlying mechanisms are currently unclear. In the present study, we found that VD3 ameliorated NM‐induced cutaneous inflammation by inactivating the NLRP3 inflammasome, which was partially mediated through the SIRT3–SOD2–mtROS signaling pathway. These results provide a new molecular mechanism of VD3 that might be utilized for the treatment of NM‐induced dermal toxicity.

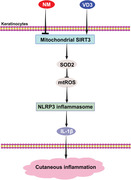

**Abbreviations**
3‐TYP: 3‐(1H‐1,2,3‐triazol‐4‐yl)pyridine
AIM2: absent in melanoma 2
Casp1 p20: activated caspase‐1 p20
COX2: cyclooxygenase‐2
DMSO: dimethyl sulfoxide
IL‐1: interleukin‐1
iNOS: inducible nitric oxide synthase
MAPK: mitogen‐activated protein kinase
mtROS: mitochondrial ROS
NF‐κB: nuclear transcription factor‐κB
NLRC4: nucleotide oligomerization domain‐like receptor C4
NLRP3: NOD‐like receptor family pyrin domain containing 3
NM: nitrogen mustard
ROS: reactive oxygen species
SIRT3: sirtuin 3
siRNA: small interference RNA
SOD2: superoxide dismutase 2
TEMPO: mito‐TEMPO
TMAO: trimethylamine‐*N*‐oxide
TNF‐α: tumor necrosis factor α
UVR: ultraviolet radiation
VD3: vitamin D3
VDR: vitamin D receptor
YVAD: zYVAD‐fmk

## 1 INTRODUCTION

Nitrogen mustard (NM) is an extremely toxic vesicant, which causes severe skin injury characterized by edema, blistering, induration, and ulcer. Accumulating evidence has revealed cutaneous inflammation as a key‐independent pathogenic factor contributing to NM‐triggered dermal toxicity, which thus presents a viable novel therapeutic target.^1^, ^2^, ^3^ Although several studies have focused on anti‐inflammatory agents against NM‐caused dermal toxicity over the past few decades, no satisfactory therapeutic outcomes have been reported to date.^4^, ^5^ One major limitation is the lack of knowledge on the specific mechanisms underlying NM‐induced cutaneous inflammation.

NOD‐like receptor family pyrin domain containing 3 (NLRP3) inflammasome is the most extensively characterized inflammasome, which activates caspase‐1 and induces interleukin‐1 (IL‐1) β release. The NLPR3 inflammasome is recently shown to be involved in the pathogenesis of diverse skin disorders and injuries.^6^ For instance, Niebuhr and co‐workers demonstrated that *Staphylococcus aureus* colonization and infection leads to NLRP3 inflammasome impairment, which contributes to chronic skin inflammation in atopic dermatitis.^7^ Another study by Watanabe et al.^8^ showed that different kinds of contact sensitizers cause contact hypersensitivity by regulating the NLRP3 inflammasome activity in keratinocytes. Ultraviolet radiation (UVR) is reported to activate the NLRP3 inflammasome, thereby leading to the secretion of IL‐1β in keratinocytes, and in turn, triggering cutaneous inflammation.^9^ Moreover, inactivation of the NLRP3 inflammasome has been implicated in the inhibition of cutaneous inflammation and consequent attenuation of skin disorders.^6^, ^10^ Therefore, the NLRP3 inflammasome is considered as a key factor in the pathogenesis of cutaneous inflammation. Additionally, NM is known to promote cutaneous inflammation through activating the mitogen‐activated protein kinase (MAPK) or nuclear transcription factor‐κB (NF‐κB) signaling pathways.^2^ However, little is known about the role of the NLRP3 inflammasome in NM‐induced cutaneous inflammation and the underlying mechanisms.

Reactive oxygen species (ROS), especially mitochondrial ROS (mtROS), represent one of the most important triggers of NLRP3 inflammasome activation.^11^, ^12^ Sirtuin 3 (SIRT3) is one of the Sir2 family and plays a critical role in maintaining mtROS homeostasis.^13^ SIRT3 is a mitochondrial deacetylase that deacetylates the antioxidant enzymes in mitochondria to regulate mtROS productions,^14^ among which superoxide dismutase 2 (SOD2) is the most important target. SIRT3 activates SOD2 through direct binding and deacetylation, thereby regulating mtROS homeostasis and NLRP3 inflammasome activation.^15^, ^16^

Vitamin D3 (VD3), a pro‐hormone synthesized in skin, shows efficacy against NM‐induced dermal toxicity. A single dose of VD3 is reported to protect against NM‐induced skin injury via suppressing macrophage‐mediated inducible nitric oxide synthase (iNOS) production.^17^ Moreover, VD3 reduces oxidative stress‐mediated inflammation predominantly induced by particulate matter through inactivating the NLRP3 inflammasome.^18^ VD3 has additionally been shown to attenuate periodontitis via inactivating NLRP3 inflammasome in the mouse model.^19^ These published data suggest the involvement of the NLRP3 inflammasome in the protective effects of VD3. However, the precise contribution of the inflammasome to VD3‐mediated protection against NM‐triggered dermal toxicity remains to be elucidated.

Data from the current study demonstrated for the first time that VD3 efficiently protected keratinocytes against NM‐induced cutaneous inflammation via inactivating the NLRP3 inflammasome, partially through the SIRT3–SOD2–mtROS signaling pathway. These results provide a new molecular mechanism of VD3 that might be utilized for treating NM‐caused dermal toxicity.

## 2 MATERIALS AND METHODS

### 2.1 Cell treatments

HaCaT cells were incubated with NM at the concentration of 0, 1, 5, 10, and 20 μM for 4 h. Cells were treated with 3‐(1H‐1,2,3‐triazol‐4‐yl)pyridine (3‐TYP; 50 μM), mito‐TEMPO (TEMPO; 50 μM), MCC950 (10 μM), or zYVAD‐fmk (YVAD; 10 μM) for 1 h, following incubation with NM (20 μM) for another 4 h in the presence or absence of 1,25(OH)2D3 (VD3, 10 nM). All the inhibitors were dissolved in dimethyl sulfoxide (DMSO) and were diluted to the working concentration with full Roswell Park Memorial Institute‐1640 medium. And the control group was treated with 0.1% DMSO.

### 2.2 Animal treatments

Eight‐week‐old female C57BL/6J and SIRT3^−/−^ mice were provided by Jackson Laboratory and maintained on a standard laboratory diet. All animal experiments were approved by the Animal Care and Use Committee of the Third Military Medical University (Chongqing, China).

Pentobarbital sodium anesthetic (50 mg/kg) was intraperitoneally injected to anesthetize mice and dorsal fur were removed with clippers and depilating cream. After 48 h, a circular area of 0.5 cm^2^ on the dorsal–lumbar region of the animal and centered on the body axis, where mice were not able to perturb, was chosen for NM exposure in a fume hood as reported previously.^20^ And dorsal skin of mice (n = 6/group) was topically exposed to NM (3.2 mg/200 μL acetone), whereas control mice received 200 μL acetone only, as described earlier.^21^ VD3 was reconstituted in DMSO and diluted in mineral oil for intraperitoneal injection at a concentration of 50 ng/100 μL per mouse combined with or without anakinra (50 mg/kg), at 1 h before NM exposure. The same volume of mineral oil was injected into the mice in the control group. All the mice were kept under anesthesia during the NM exposure. At 4 h after NM exposure, exposed regions of skin were gently wiped with sodium hypochlorite (0.8%) and saline for decontamination and then were returned to the animal house each in a single cage. Images of wounds were obtained with a digital camera on day 1, 3, 7, and 12. Mice were sacrificed at day 3 or 12 following NM exposure. We collected the dorsal skin tissues and fixed them in 10% formalin for histopathological analysis or snap‐frozen in liquid nitrogen for western blot analysis.

**Highlights**

1. NLRP3 inflammasome played a critical role in NM‐induced cutaneous inflammation;
2. NM activated NLRP3 inflammasome through the SIRT3–SOD2–mtROS signaling pathway;
3. Targeting SIRT3 by VD3 ameliorated NM‐induced cutaneous inflammation via inactivating the NLRP3 inflammasome.

Full descriptions of additional materials and methods are given in the Supporting Information.

## 3 RESULTS

### 3.1 NM induced inflammation in keratinocytes

**FIGURE 1:**
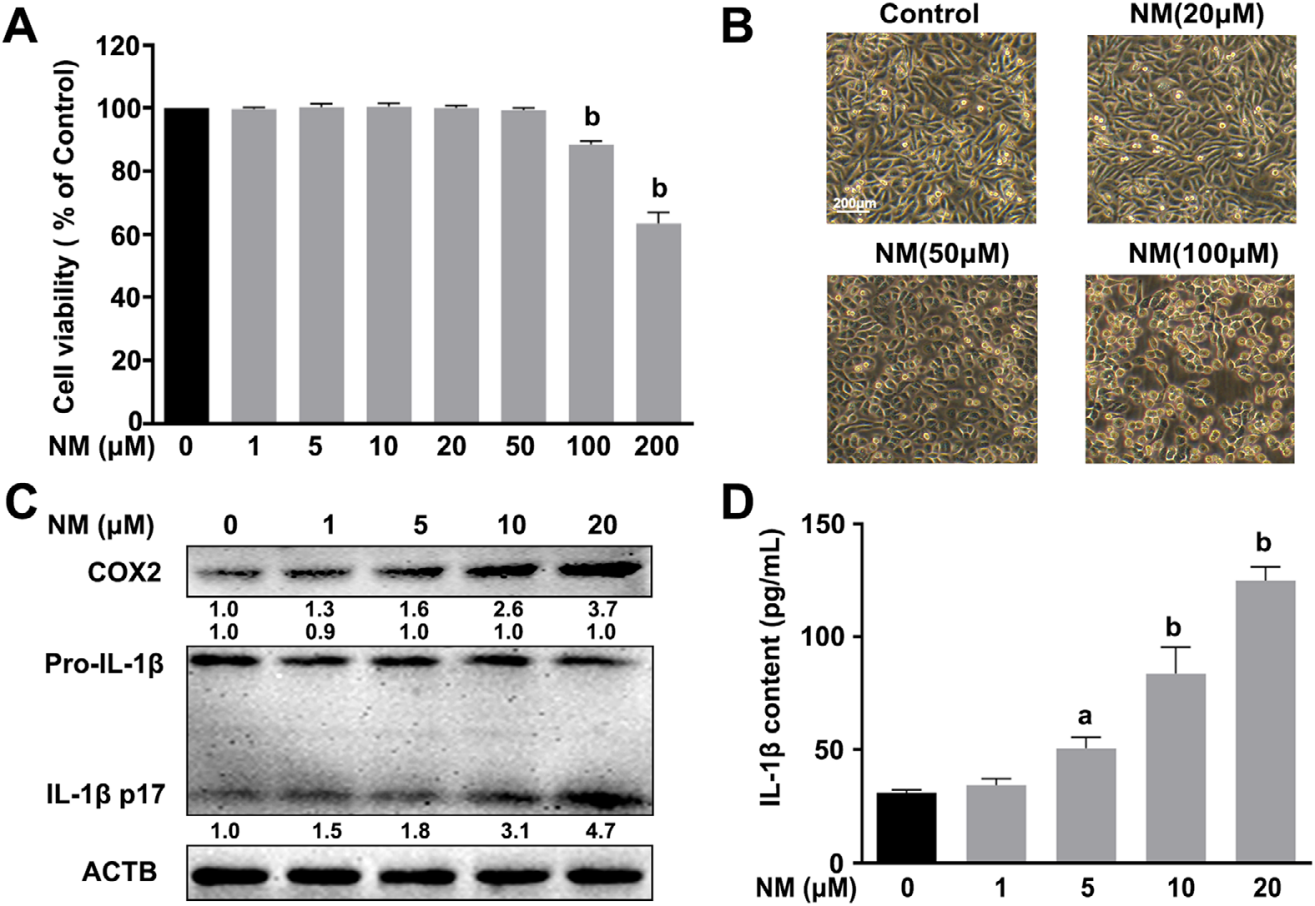
NM induced inflammation in keratinocytes. (**A**) HaCaT cells were incubated with different concentrations of NM (0, 1, 5, 10, 20, 50, 100, and 200 μM) for 4 h and cell viability was determined as described in Section 2. **(B)** Cells were treated with NM (0, 20, 50, and 100 μM) for 4 h and morphological characteristics were examined via microscopy. **(C)** Cells were treated with different concentrations of NM (0, 1, 5, 10, and 20 μM) for 4 h; pro‐IL‐1β, IL‐1β p17, and COX2 expression were determined via western blot. The representative immunoblots were quantified by densitometric analysis. (**D**) Cells were treated as described in panel C and the IL‐1β content in the supernatant fraction was analyzed via ELISA. Values are presented as means ± SD (*n* = 3). ^a^ *p* < 0.05 and ^b^ *p* < 0.01 versus vehicle‐treated control group (the black bar)

To examine the cytotoxicity of NM to keratinocytes, HaCaT cells were incubated with various concentrations of NM (0, 1, 5, 10, 20, 50, 100, and 200 μM) for 4 h and cell viability was detected. NM at concentrations of up to 50 μM had no obvious effects on cell viability and morphology (Figures 1A and 1B). Cyclooxygenase‐2 (COX2; an enzyme that acts to speed up the formation of prostanoids) is widely used as an inflammatory marker of skin inflammation, which is also necessary in the pathogenesis of NM‐caused cutaneous injury.^22^, ^23^ Therefore, we measured the expression of COX2 in NM‐treated keratinocytes. As shown in Figures 1C and 1D, COX2 expression and IL‐1β secretion were significantly induced by NM at concentrations ≤20 μM. The results indicated that relatively low concentrations of NM (≤20 μM) can trigger inflammation with no obvious effects on cell viability in keratinocytes. Accordingly, NM was used at a concentration of 20 μM in subsequent experiments to clarify the mechanisms underlying cutaneous inflammation.

### 3.2 NM induced inflammation via activating the NLRP3 inflammasome in keratinocytes

**FIGURE 2:**
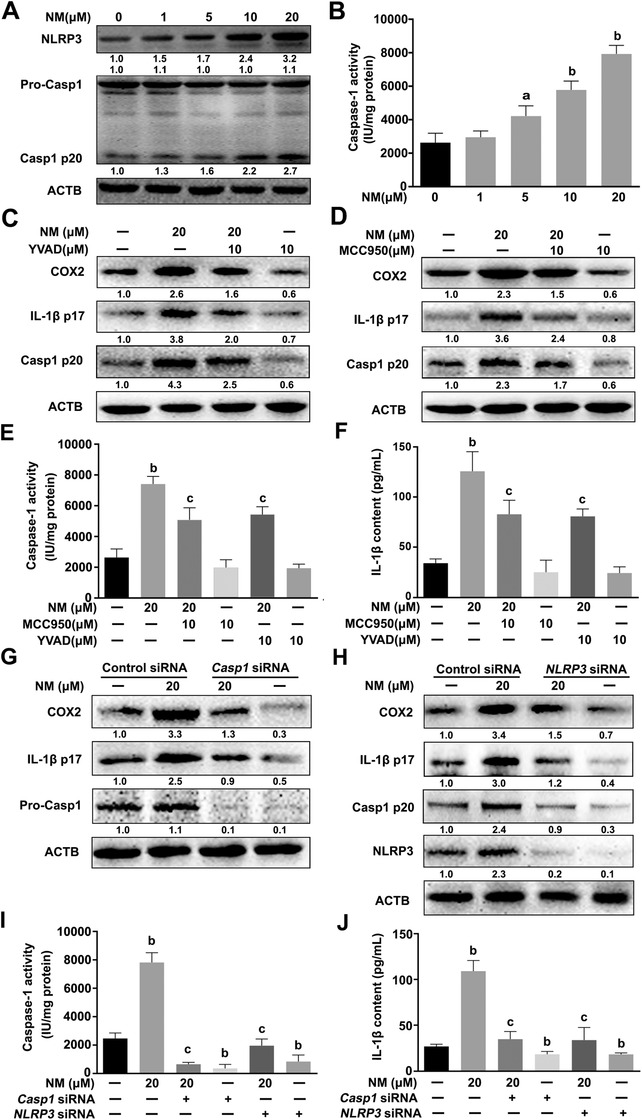
NM induced inflammation via activating the NLRP3 inflammasome in keratinocytes. NM induced activation of the NLRP3 inflammasome in keratinocytes. HaCaT cells were treated with various concentrations of NM (0, 1, 5, 10 and 20 μM) for 4 h. **(A)** Western blot analysis of NLRP3, pro‐caspase 1 (pro‐Casp1), and Casp1 p20 levels. **(B)** Measurement of caspase‐1 activity with a specific caspase‐1 activity kit. Cells were pretreated with YVAD (10 μM) or MCC950 (10 μM) for 1 h and exposed to NM (20 μM) for a further 4 h. **(C** and **D)** Western blot analysis of COX2, IL‐1β p17, and Casp1 p20 expression. **(E)** Caspase‐1 activity was measured using a caspase‐1 activity kit. (**F**) ELISA analysis of IL‐1β secretion. HaCaT cells were transfected with *caspase‐1* siRNA or *NLRP3* siRNA as described in Section 2. After 24 h, cells were incubated with NM (20 μM) for 4 h. **(G** and **H)** The expression of COX2, IL‐1β p17, pro‐Casp1, Casp1 p20, and NLRP3 was detected via western blot. **(I)** Caspase‐1 activity was measured using a caspase‐1 activity kit. (**J**) ELISA analysis of IL‐1β secretion. All the representative immunoblots were quantified by densitometric analysis. Values are expressed as means ± SD (n = 3). ^a^ *p* < 0.05 and ^b^ *p* < 0.01 versus vehicle‐treated control group (the black bar); ^c^ *p* < 0.05 versus single NM‐treated group

Next, we examined the effect of NM on NLRP3 inflammasome activity in keratinocytes. As shown in Figures 2A and 2B, NM had no significant effect on pro‐caspase 1 expression, but markedly upregulated the expression of NLRP3 and caspase‐1 p20 (casp1 p20) and increased caspase‐1 activity in keratinocytes. Keratinocytes were exposed to NM with MCC950 (a selective NLRP3 inhibitor) or YVAD (a specific caspase‐1 inhibitor) to further define the potential involvement of the NLRP3 inflammasome in NM‐caused inflammation in keratinocytes. As indicated in Figures 2C–F, NM‐induced caspase‐1 activation, IL‐1β secretion, and COX2 expression were notably abolished by MCC950 (10 μM) or YVAD (10 μM) in keratinocytes. Transfection with *caspase‐1* or *NLRP3* small interference RNA (siRNA) also inhibited NM‐induced NLRP3, casp1 p20, IL‐1β p17, and COX2 expression (Figures 2G and 2H). Meanwhile, NM‐caused caspase‐1 activation and IL‐1β release were also abolished by *caspase‐1* or *NLRP3* siRNA transfection (Figures 2I and 2J). Additionally, NM had no significant effect on absent in melanoma 2 (AIM2) or nucleotide oligomerization domain‐like receptor C4 (NLRC4) expression, indicating that NM could not activate these types of inflammasome in keratinocytes (Figure S1). The collective findings indicated that the NLRP3 inflammasome played a critical role in NM‐induced inflammation in keratinocytes.

### 3.3 mtROS mediated NM‐induced NLRP3 inflammasome activation in keratinocytes

**FIGURE 3:**
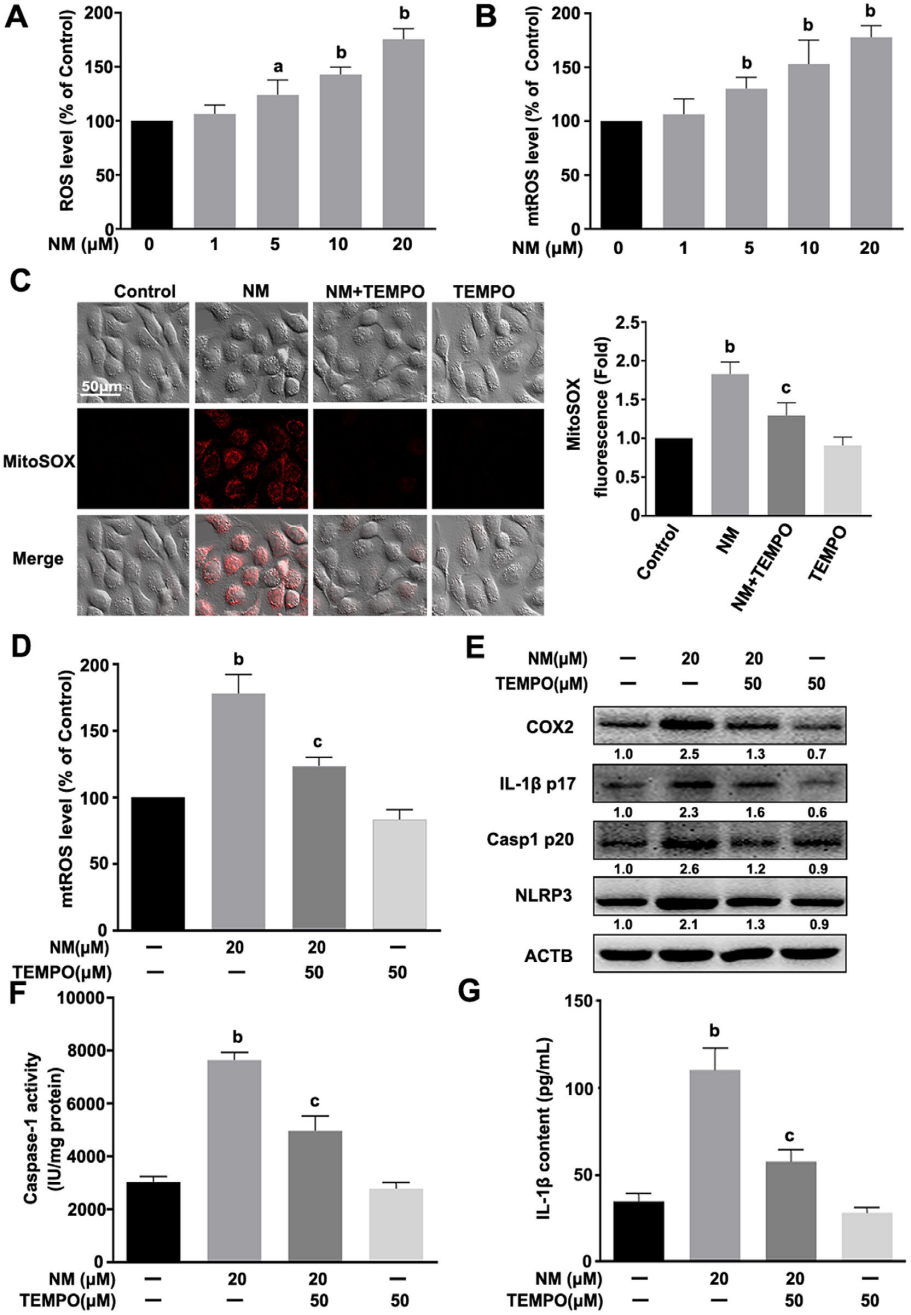
mtROS mediated NM‐induced NLRP3 inflammasome activation in keratinocytes. HaCaT cells were treated with various concentrations of NM (0, 1, 5, 10, and 20 μM) for 4 h. **(A)** Detection of total ROS levels using DCFH‐DA. **(B)** Detection of mtROS levels with MitoSOX^TM^ Red as described in Section 2. Cells were pretreated with TEMPO (50 μM) for 1 h, followed by NM (20 μM) for another 4 h. **(C)** Representative images of mtROS detected using a ZEISS LSM800 confocal laser scanning microscope. **(D)** Quantification of mtROS levels using a fluorescence spectrometer. **(E)** Total cell lysates were immunoblotted with anti‐COX2, anti‐IL‐1β p17, anti‐Casp1 p20, anti‐NLRP3 or anti‐β‐actin (ACTB) antibodies. And the representative immunoblots were quantified by densitometric analysis. **(F)** Analysis of caspase‐1 activity using a specific assay kit. **(G)** ELISA analysis of IL‐1β secretion in cell culture supernatant fractions. Values are expressed as means ± SD (n = 3). ^a^ *p* < 0.05 and ^b^ *p* < 0.01 versus vehicle‐treated control group (the black bar); ^c^ *p* < 0.05 versus single NM‐treated group.

Previous studies have identified mtROS as an important trigger of NLRP3 inflammasome activation.^11^ Accordingly, we focused on the potential involvement of mtROS in NM‐induced NLRP3 inflammasome activation. As indicated in Figures 3A and 3B, NM markedly increased the levels of total ROS and mtROS in keratinocytes. Moreover, pretreatment with TEMPO (a specific mtROS scavenger) inhibited mtROS production and NLRP3 inflammasome activation mediated by NM, thereby decreasing IL‐1β p17 and COX2 levels (Figures 3C–G). Our data validated the requirement of mtROS for this process.

### 3.4 NM induced mtROS accumulation via the SIRT3–SOD2 pathway in keratinocytes

**FIGURE 4:**
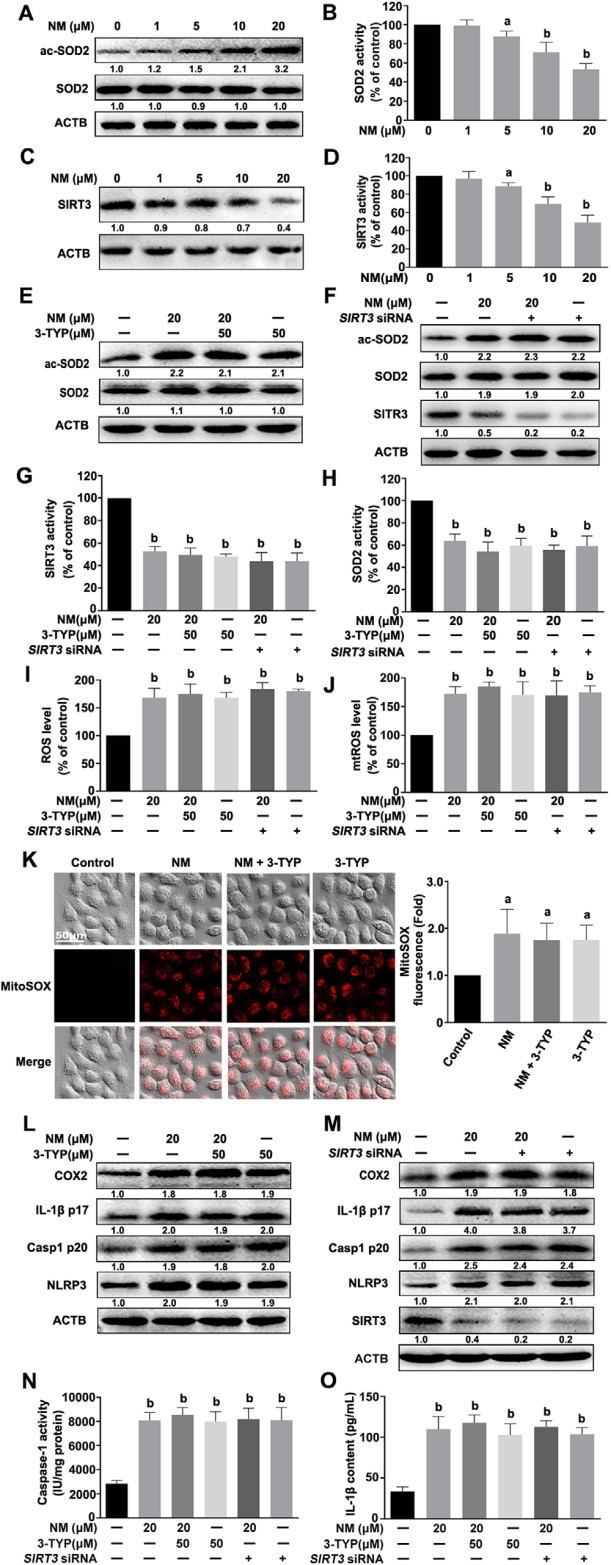
NM induced mtROS accumulation via the SIRT3–SOD2 pathway in keratinocytes. Cells were treated with various concentrations of NM (0, 1, 5, 10, and 20 μM) for 4 h. **(A)** Western blot analysis of acetylation‐SOD2 (ac‐SOD2) and SOD2 expression. **(B)** Assay of SOD2 enzymatic activity using a specific kit following the manufacturer's instructions. **(C)** Western blot analysis of SIRT3 expression. **(D)** Assay of SIRT3 activity using a specific kit following the manufacturer's instructions. HaCaT cells were pretreated with 3‐TYP (50 μM) for 1 h or *SIRT3* siRNA, as described in Section 2, and incubated with NM (20 μM) for a further 4 h. **(E** and **F)** Western blot analysis of ac‐SOD2, SOD2, and SIRT3 expression. **(G)** Assay of SIRT3 activity with a specific SIRT3 kit following the manufacturer's instructions. **(H)** Assay of SOD2 activity using a specific kit following the manufacturer's instructions. **(I)** Detection of total ROS levels using DCFH‐DA or **(J)** mtROS levels using MitoSOX^TM^ Red, followed by measurement on an Infinite^TM^ M200 Microplate Reader. **(K)** Representative images of mtROS detected using a ZEISS LSM800 confocal laser scanning microscope. **(L** and **M)** Western blot analysis of COX2, IL‐1β p17, Casp1 p20, NLRP3, and SIRT3 expression. **(N)** Assay of caspase‐1 activity with a specific kit following the manufacturer's instructions. **(O)** ELISA evaluation of IL‐1β secretion in cell culture supernatant. All the representative immunoblots were quantified by densitometric analysis. Values are expressed as means ± SD (n = 3). ^a^ *p* < 0.05 and ^b^ *p* < 0.01 versus vehicle‐treated control group (the black bar)

The activity of SOD2, a crucial scavenger of mtROS,^24^ was monitored in NM‐treated keratinocytes. As shown in Figures 4A and 4B, the SOD2 activity was dose‐dependently inhibited by NM. The mitochondrial sirtuin, SIRT3, has been identified as the major regulator of SOD2 that acts through direct binding and deacetylation of specific conserved lysine residues.^15^ As expected, NM notably inhibited SIRT3 expression and activity (Figures 4C and 4D). The findings revealed that NM suppressed both SIRT3 and SOD2 activity.

Furthermore, 3‐TYP (a selected inhibitor of SIRT3) or *SIRT3* siRNA was also employed to ascertain the possible role of the SIRT3–SOD2 pathway in mtROS accumulation in NM‐exposed keratinocytes. As shown in Figures 4E–K, in the presence of 3‐TYP or *SIRT3* siRNA, NM was not able to further inhibit SIRT3 or SOD2 activities or increase total ROS or mtROS levels in keratinocytes. Meanwhile, NM also failed to further induce NLRP3 inflammasome activation, IL‐1β release, and COX2 expression in keratinocytes co‐treated with 3‐TYP or *SIRT3* siRNA (Figures 4L–O). Our collective data supported the requirement of the SIRT3–SOD2 pathway for mtROS accumulation and subsequent activation of the inflammasome in NM‐exposed keratinocytes.

### 3.5 VD3 protected keratinocytes against NM‐induced inflammation via inhibiting the NLRP3 inflammasome

**FIGURE 5:**
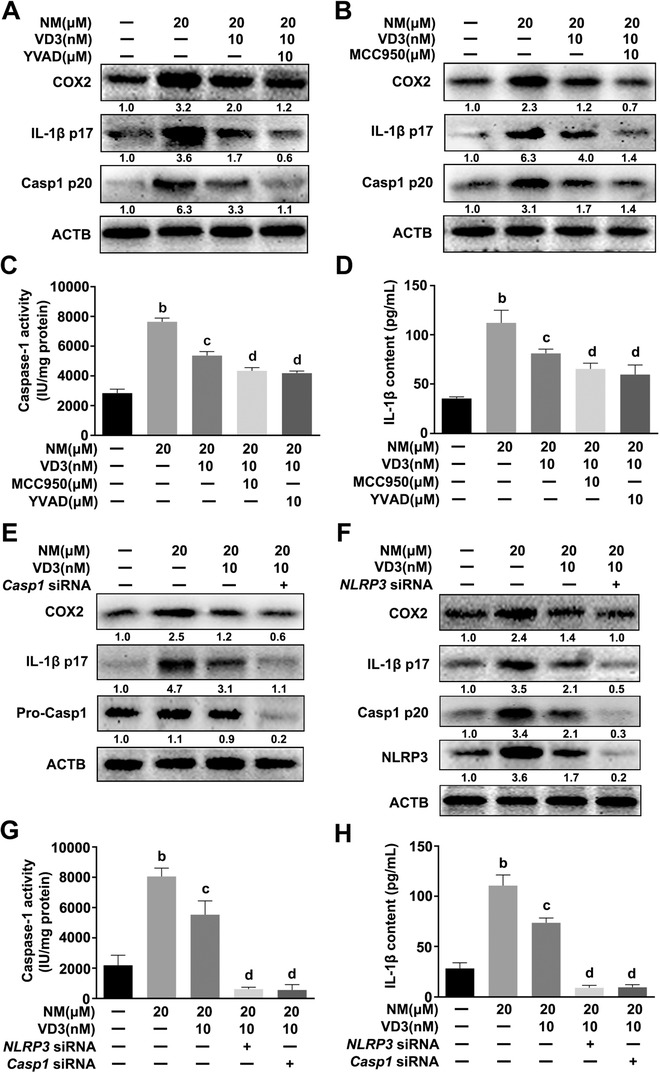
VD3 protected keratinocytes against NM‐induced inflammation via inhibiting the NLRP3 inflammasome. HaCaT cells were pretreated with VD3 (10 nM) in the presence or absence of YVAD (10 μM) or MCC950 (10 μM) for 1 h, followed by treatment with NM (20 μM) for another 4 h. **(A** and **B)** Western blot analysis of Casp1 p20, COX2, and IL‐1β p17 contents. **(C)** Assay of caspase‐1 activity with a specific kit following the manufacturer's instructions. **(D)** ELISA measurement of IL‐1β secretion in cell culture supernatant. HaCaT cells were transfected with **(E)** *caspase‐1* siRNA or **(F)** *NLRP3* siRNA, as described in Section 2. After 24 h, cells were incubated with NM (20 μM) for 4 h, and the expression of COX2, IL‐1β p17, pro‐caspase 1 (pro‐Casp1), Casp1 p20, and NLRP3 was detected via western blot. **(G)** Assay of caspase‐1 activity with a specific kit following the manufacturer's instructions. **(H)** ELISA measurement of IL‐1β secretion in cell culture supernatant. All the representative immunoblots were quantified by densitometric analysis. Values are expressed as means ± SD (n = 3). ^b^ *p* < 0.01 versus vehicle‐treated control group (the black bar); ^c^ *p* < 0.05 versus single NM‐treated group; ^d^ *p* < 0.05 versus NM and VD3 co‐treated group

Recently, VD3 was shown to accelerate wound healing following NM‐induced skin injury in mice;^17^ however, the exact mechanisms remain to be explored. According to our above findings, the role of the NLRP3 inflammasome in VD3‐mediated protection against NM‐induced dermal toxicity was also investigated. As shown in Figures 5A–H, S2A, S3A, and S3B, VD3 dose‐dependently attenuated NM‐induced NLRP3, Casp1 p20, IL‐1β p17, and COX2 expression, caspase‐1 activation, and the release of IL‐1β, IL‐6, and tumor necrosis factor α (TNF‐α). Moreover, these effects were enhanced when NLRP3 or caspase‐1 was suppressed using selective inhibitors or siRNAs (Figures 5A–H, S3A, and S3B). These results clearly indicated that VD3 attenuated NM‐triggered cutaneous inflammation in a NLRP3 inflammasome‐dependent manner.

### 3.6 VD3 suppressed NM‐induced NLRP3 inflammasome activation via the SIRT3–SOD2–mtROS signaling pathway in keratinocytes

**FIGURE 6:**
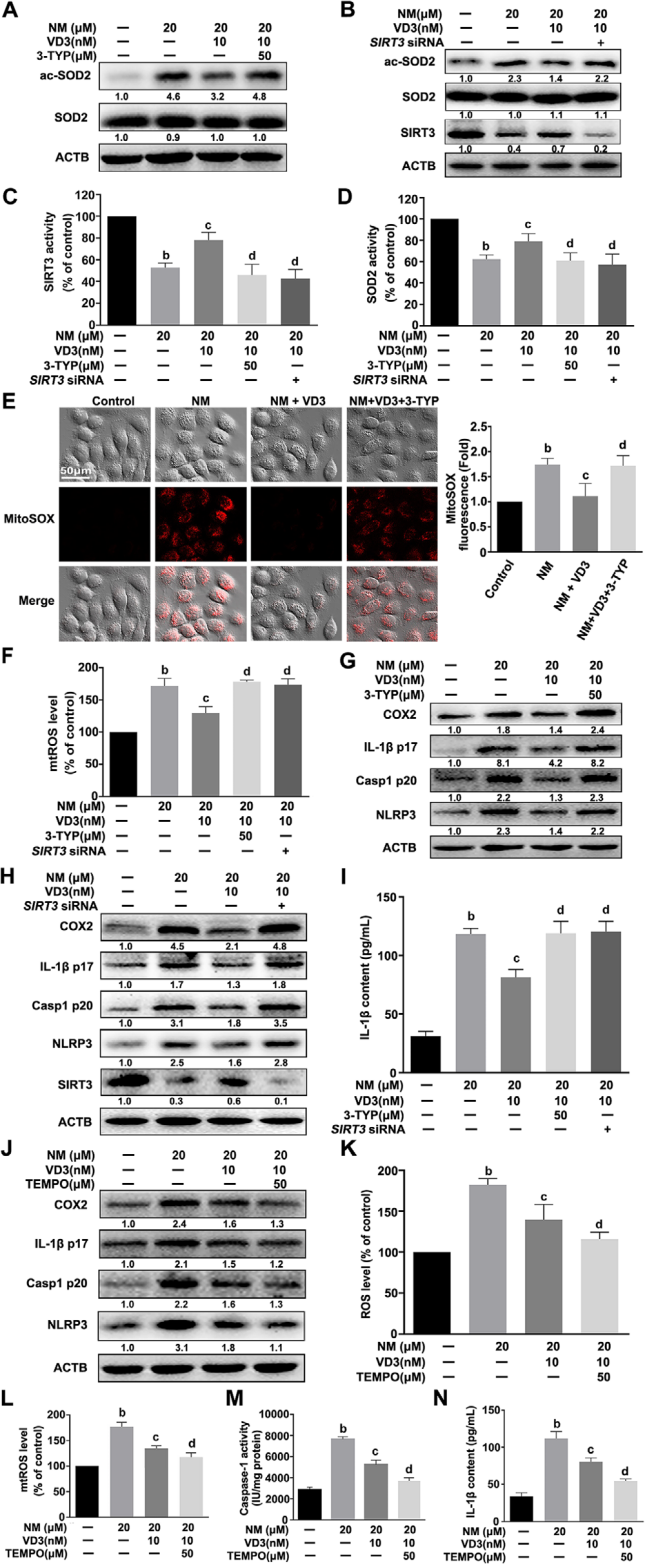
VD3 suppressed NM‐induced NLRP3 inflammation activation through the SIRT3–SOD2–mtROS pathway in keratinocytes. **(A)** HaCaT cells were pretreated with VD3 (10 nM) in the presence or absence of 3‐TYP (50 μM) for 1 h, followed by treatment with NM (20 μM) for another 4 h. The expression of acetylation‐SOD2 (ac‐SOD2) and SOD2 was detected by western blot. **(B)** SIRT3 was eliminated via transfection with *SIRT3* siRNA, as described in Section 2. At 24 h posttransfection, cells were treated with VD3 (10 nM) for 1 h and then incubated with NM (20 μM) for an additional 4 h. Cells were collected and lysed and subjected to western blot. Cells were treated as described in panels A and B. **(C)** The activity of SIRT3 was determined by SIRT3 Assay Kits. **(D)** SOD2 activity was measured by SOD2 Assay Kits. **(E)** Cells were treated as described in panel A. Afterward, the cells were incubated with MitoSOX^TM^ Red (5 μM, 10 min, at 37°C) and immediately visualized by confocal microscopy. HaCaT cells were treated as described in panels A and B. **(F)** mtROS levels were detected with MitoSOX™ Red by an Infinite^TM^ M200 Microplate Reader. **(G** and **H)** Western blot analysis of COX2, IL‐1β p17, Casp1 p20, NLRP3, and SIRT3 expression. **(I)** IL‐1β secretion in cell culture supernatant was measured via ELISA. HaCaT cells were pretreated with VD3 (10 nM) in the presence or absence of TEMPO (50 μM) for 1 h, followed by the incubation of NM (20 μM) for an additional 4 h. **(J)** Western blot analysis of COX2, IL‐1β p17, Casp1 p20, and NLRP3 expression. **(K)** Detection of total ROS levels using DCFH‐DA. **(L)** Detection of mtROS levels with MitoSOX™ Red. **(M)** Assay of caspase‐1 activity with a specific kit following the manufacturer's instructions. **(N)** ELISA analysis of IL‐1β secretion in cell culture supernatant fractions. All the representative immunoblots were quantified by densitometric analysis. Values are expressed as means ± SD (n = 3). ^b^ *p* < 0.01 versus vehicle‐treated control group (the black bar); ^c^ *p* < 0.05 versus single NM‐treated group; ^d^ *p* < 0.05 versus NM and VD3 co‐treated group

We have identified that SIRT3 is a key inhibitor of NM‐induced inflammasome activation, and accordingly the potential relationship between SIRT3 and VD3 in keratinocytes was examined. As expected, VD3 restored SIRT3 expression and SIRT3 and SOD2 activities and reduced acetylation‐SOD2 expression and mtROS production in NM‐treated keratinocytes (Figures 6A–F and S2B). 3‐TYP or *SIRT3* siRNA pretreatment abolished the effect of VD3 on NM‐stimulated keratinocytes (Figures 6A–F), thereby attenuating the VD3‐induced decrease in NLRP3, IL‐1β p17, Casp1 p20, and COX2 expression and IL‐1β secretion (Figures 6G–I). Our results highlighted the involvement of SIRT3–SOD2 in the protective effect of VD3 against NM‐induced inflammasome activation in keratinocytes.

Moreover, TEMPO pretreatment significantly enhanced VD3‐induced inhibition of total ROS and mtROS production, NLRP3 and COX2 expression, caspase‐1 activation, and IL‐1β release in NM‐stimulated keratinocytes (Figures 6J–N), suggesting that mtROS was essential in VD3‐mediated inhibition of the NLRP3 inflammasome. Thus, we concluded that the SIRT3–SOD2–mtROS pathway was important for VD3‐induced NLRP3 inflammation inhibition in NM‐treated keratinocytes.

### 3.7 VD3 attenuated NM‐induced dermal toxicity by inactivating NLRP3 inflammasome through SIRT3–SOD2 pathway in vivo

**FIGURE 7:**
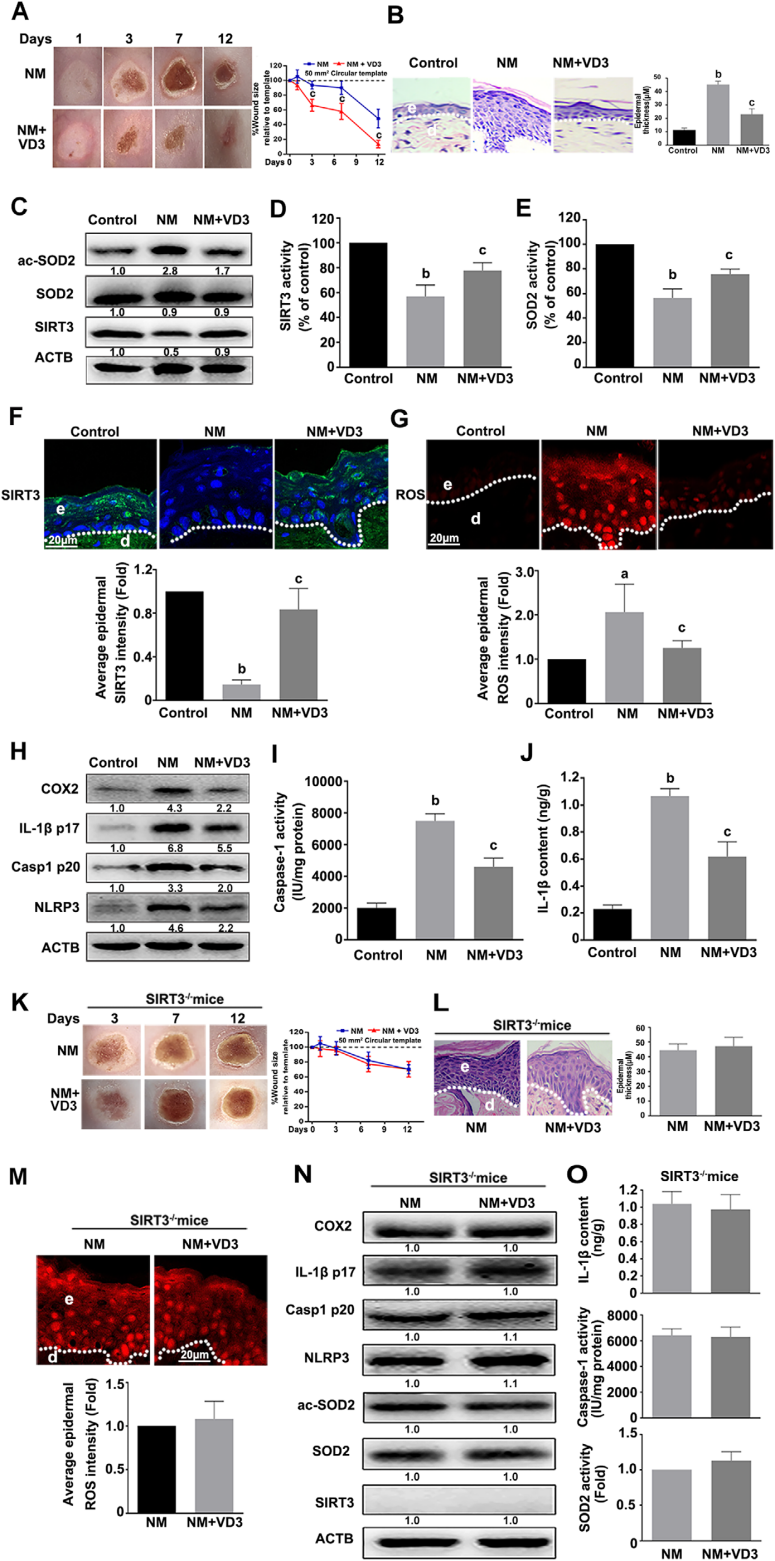
VD3 suppressed NM‐induced NLRP3 inflammasome activation through the SIRT3–SOD2 pathway in vivo. Dorsal skins of 8‐week‐old female C57BL/6J mice (n = 6 per group) were exposed to 3.2 mg NM in 200 μL acetone in the presence or absence of VD3 (50 ng/100 μL, i.p.), as described in Section 2. **(A)** Representative images of skin wound healing post‐NM exposure. Mice were sacrificed at day 12 following NM exposure, and dorsal skin tissue was collected. **(B)** H&E staining was performed to analyze injured skin (400× magnification). **(C)** Western blot analysis of acetylation‐SOD2(ac‐SOD2), SOD2, and SIRT3 expression. **(D)** Measurement of SIRT3 activity and **(E)** SOD2 activity from NM‐exposed skin. At day 3 after NM exposure, the skin wounds and nearby tissues were collected. **(F)** SIRT3 expression was detected by immunofluorescence analysis and **(G)** confocal laser scanning microscope observation of ROS levels in the frozen sections stained with dihydroethidium (DHE). Mice were sacrificed at day 12 following NM exposure, and dorsal skin tissue was collected. **(H)** Western blot analysis of COX2, IL‐1β p17, Casp1 p20, and NLRP3 expression. (**I)** Assay of caspase‐1 activity using a specific kit following the manufacturer's instructions. **(J)** ELISA analysis of IL‐1β contents. Dorsal skins of 8‐week‐old female SIRT3^−/−^ mice (n = 6 per group) were exposed to 3.2 mg NM in 200 μL acetone in the presence or absence of VD3 (50 ng/100 μL, i.p.), as described in Section 2. **(K)** Representative images of skin wound healing post‐NM exposure. Mice were sacrificed at day 12 following NM exposure, and dorsal skin tissue was collected. **(L)** H&E staining was performed to analyze injured skin (400× magnification). **(M)** At day 3 after NM exposure, the skin wounds and nearby tissues were collected. The ROS levels were detected with DHE staining followed by confocal laser scanning microscope analysis. **(N)** Western blot analysis of COX2, IL‐1β p17, Casp1 p20, NLRP3, ac‐SOD2, SOD2, and SIRT3 expression. **(O)** ELISA analysis of IL‐1β contents (top). Assay of caspase‐1 activity using a specific kit following the manufacturer's instructions (middle). SOD2 activity from NM‐exposed skin (bottom). All the representative immunoblots were quantified by densitometric analysis. e, epidermis; d, dermis. Values are expressed as means ± SD (n = 6). ^a^ *p* < 0.05 and ^b^ *p* < 0.01 versus vehicle‐treated control group (the black bar); ^c^ *p* < 0.05 versus single NM‐treated group

To ascertain whether VD3‐mediated suppression of NM‐induced cutaneous inflammation involves a similar mechanism in vivo, an NM‐exposed skin injury mouse model was used. In line with the previous published work,^17^ VD3 accelerated wound healing and promoted maturation of new epidermis, as evident from examination of the epidermal thickness of NM‐exposed skin (Figures 7A and 7B). VD3 induced a significant increase in SIRT3 and SOD2 activities, thereby inhibiting ROS generation; NLRP3, Casp1 p20, IL‐1β p17, and COX2 expression; caspase‐1 activation; and IL‐1β release in NM‐exposed skin (Figures 7C–J). Moreover, anakinra (an IL‐1 receptor antagonist) enhanced VD3‐induced improvement of skin wound healing in NM‐exposed mice (Figures S4A and S4B). In addition, the beneficial effects of VD3 on NM‐caused dermal toxicity, ROS generation, and NLRP3 inflammasome activation were abolished in SIRT3^−/−^ mice (Figures 7H–O). These data indicated that SIRT3 played a critical role in VD3‐induced SOD2 activation, ROS generation, and NLRP3 inflammasome inhibition and subsequent attenuation of cutaneous inflammation caused by NM in vivo.

## 4 DISCUSSION

Cutaneous inflammation has been identified as an initial step in NM‐induced skin injury. Earlier studies have shown that MAPK and NF‐κB signaling pathways were involved in vesicants including NM‐induced inflammatory cytokines production in skin.^2^ p38 MAPK inhibition is reported to downregulate sulfur mustard‐induced cytokine release in keratinocytes.^25^ Recently, our group showed that NM prevent fos‐related antigen‐1 from entering into the nucleus, which leads to IL‐8 overexpression and contributes to NM‐induced cutaneous inflammation.^20^ Anti‐inflammatory agents targeting these related signaling pathways have been widely explored for the treatment of vesicant‐caused dermal toxicity.^26^ However, effective targeted therapies are still inadequate, suggesting the involvement of other specific mechanisms in vesicant‐induced cutaneous inflammation.

Data from the current study demonstrated for the first time that NM induces cutaneous inflammation by activating the NLRP3 inflammasome. The extensively characterized NLRP3 inflammasome is assembled and activated by numerous environmental irritants and endogenous danger signals.^27^ The NLRP3 inflammasome activation leads to caspase‐1‐mediated release of IL‐1β that plays a critical role in inflammation. The NLRP3 inflammasome contributes to host defense under normal physiological conditions^28^; however, excessive activation evokes inflammatory diseases, especially skin disorders.^29^ Researchers have confirmed that the NLRP3 inflammasome activation is involved in contact hypersensitivity and inhibition of NLRP3 inflammasome signaling presents a potential strategy for prevention of allergic contact dermatitis.^30^ Niebuhr et al.^7^ demonstrated that the Th2 milieu causes impairment of NLRP3 inflammasome expression and function, leading to atopic dermatitis, a chronic inflammatory skin disorder. Moreover, UVR induces sunburn and inflammation in the epidermis by promoting IL‐1β release in a NLRP3 inflammasome‐dependent manner.^31^ NLRP3 inflammasome‐related signaling deficiency is additionally reported to attenuate UVR‐triggered dermal toxicity in cell and rodent models.^9^, ^31^ These results clearly support that the NLRP3 inflammasome was necessary for the pathogenesis of cutaneous inflammation and consequent development of skin disorders. Data from our study indicated that NM activated NLRP3 inflammasome and increased IL‐1β release, thereby causing cutaneous inflammation. Meanwhile, we also found that NM could not activate AIM2 or NLRC4 inflammasome in keratinocytes. These findings provide further evidence of NM‐induced dermal toxicity in a NLRP3 inflammasome‐dependent manner, indicating that NLRP3 inflammasome would be a potential target for the treatment of NM‐caused dermal toxicity. Moreover, it has been demonstrated that vesicants‐caused injury elicits an inflammatory response characterized by infiltration of neutrophils and macrophages.^32^ Vesicants exposure increases both macrophage‐derived chemokines (monocyte chemoattractant protein 1 and growth‐regulated oncogene α) and neutrophil‐derived chemokines (macrophage inflammatory protein 1α and IFN‐γ‐inducible protein 10), which might further contribute to the pathogenesis of vesicants‐induced skin injury.^32^ Meanwhile, the IL‐1β release in neutrophils has recently been found to play a key role in the pathogenesis of neutrophilic dermatoses.^33^ However, whether the infiltrating neutrophils contribute to IL‐1β release in NM‐exposed skin needs further elucidation.

ROS have been identified as a critical trigger of NLRP3 inflammasome formation. Common environmental pollutants, including acroleina,^34^ cadmium,^35^ and chromium compounds,^36^ activate the NLRP3 inflammasome through elevation of ROS levels, which are abolished by ROS scavengers. Intracellular ROS are predominantly generated by mitochondria. Several studies have shown that rotenone and antimycin A promote mtROS generation, resulting in activation of the NLRP3 inflammasome.^37^, ^38^ Trimethylamine‐N‐oxide (TMAO; an independent risk factor for atherosclerosis)^39^ and chlorpyrifos (a widely used pesticide)^40^ have been shown to activate NLRP3 inflammasome in a mtROS‐dependent manner in endothelial cells and keratinocytes, respectively. Herein, we found that NM markedly increased total ROS and mtROS levels in keratinocytes. Upon scavenging of mtROS with TEMPO, NM‐induced activation of NLRP3 inflammasome was significantly inhibited, accompanied by attenuation of NM‐induced cutaneous inflammation. Although mtROS are reported to play a critical role in the activation of NLRP3 inflammasome in several skin disorders induced by other irritants, our data indicated for the first time that mtROS were critical for NLRP3 inflammasome activation in NM‐stimulated keratinocytes. This study provided important complementary findings to previous investigations about the effect of mtROS and NLRP3 inflammasome on cutaneous inflammation, highlighting potential targets for effective treatment of inflammation‐related skin disorders.

We further demonstrated the involvement of the SIRT3–SOD2‐linked pathway mtROS generation and NLRP3 inflammasome activation in NM‐treated keratinocytes. SIRT3 resides within mitochondria and maintains mitochondrial function, particularly mtROS homeostasis, through deacetylation of mitochondrial proteins, including SOD2.^13^ SOD2 is a kind of iron/manganese superoxide dismutase that plays an important role in regulating superoxide by‐products from oxidative phosphorylation.^41^ Accumulating evidence supports critical roles of SOD2 in the pathogenesis of various disorders, including diabetes,^42^ neurogenerative diseases,^43^ and skin aging,^44^ through the regulation of mtROS homoeostasis. Specifically, SIRT3 activates SOD2 through direct binding and deacetylation of specific conserved lysine residues, thereby reducing mtROS production, which exerts significant protective effects on systemic sclerosis^45^ and UVR^46^, ^47^ induced skin damage. Recently, Traba et al.^16^ confirmed that prolonged fasting suppresses NLRP3 inflammasome assembly and activation via blunting of the mtROS levels, which is mediated by the activation of SIRT3–SOD2 pathway. Liu and colleagues^48^ reported that paraquat induces a significant decrease in SIRT3 expression with subsequent inhibition of SOD2 activity, resulting in increase of mtROS levels, activation of the NLRP3 inflammasome, and ultimately, liver injury. Our previous findings additionally indicated that TMAO activates the NLRP3 inflammasome by upregulating the mtROS content through inhibition of the SIRT3–SOD2 pathway in endothelial cells.^39^ Experiments from the current study showed that NM inhibited SIRT3 and SOD2 activities. Notably, NM was not able to further decrease SOD2 activity or the NLRP3 inflammasome activation or cause inflammation in the presence of 3‐TYP or *SIRT3* siRNA in keratinocytes, supporting that the SIRT3–SOD2 pathway was involved in NM's effects on NLRP3 inflammasome activation and subsequent cutaneous inflammation. Our results implicated a potential SIRT3‐dependent strategy in attenuation of the NLRP3 inflammasome activation that can be modulated to alleviate NLRP3‐linked inflammation.

Finally, our results support an important role for the SIRT3–SOD2–mtROS signaling pathway and NLRP3 inflammasome in the protective effect of VD3 against NM‐induced dermal toxicity. VD3 is a ubiquitous fat‐soluble hormone produced in skin that contributes to calcium homeostasis and bone metabolism.^49^ Accumulating evidence supports critical roles of VD3 in the growth and differentiation of skin. Therefore, maintenance of the VD3 serum concentration within normal levels is warranted in numerous dermatological disorders, including atopic dermatitis, psoriasis, and vitiligo.^50^ In a recent study by Scott et al.,^17^ intervention with a single dose of VD3 rapidly attenuated NM‐induced cutaneous inflammation in a mouse model via inhibition of iNOS and TNF‐α release by activated macrophages. Arroyo and co‐workers^51^ additionally showed that VD3 suppresses inflammatory mediators and enhances cell proliferation in sulfur mustard‐stimulated human skin cells. These results validate the utility of VD3 as a potentially effective candidate for the treatment of cutaneous inflammation disorders caused by vesicants, but the exact mechanisms need further elucidation. Our results demonstrated that VD3 ameliorated NM‐induced cutaneous inflammation and NLRP3 inflammasome activation in vitro and in vivo. Inhibitors or siRNAs of NLRP3 and caspase‐1 notably enhanced the anti‐inflammatory effect of VD3 in NM‐treated keratinocytes. Meanwhile, VD3 ameliorated the NM‐induced increase in mtROS content. The presence of TEMPO markedly improved VD3‐induced inactivation of the NLRP3 inflammasome in NM‐treated keratinocytes. Moreover, VD3 also attenuated NM‐induced inhibition of SIRT3 and SOD2 activity. VD3's effects on NM‐induced SOD2 inhibition, mtROS generation, and NLRP3 inflammasome activation were blocked in keratinocytes treated with 3‐TYP or *SIRT3* siRNA and in skins from SIRT3^−/−^ mice. Previously, no effect of VD3 on SIRT3 expression in cardiomyoblast H9c2 cells was reported.^52^ Herein, we found that VD3 attenuated NM‐induced cutaneous inflammation by activating SIRT3 in vitro and in vivo. Although the precise mechanisms by which VD3 induces SIRT3 expression and activity remain to be established, our present data indicated that VD3 is a feasible treatment option for NM‐induced cutaneous inflammation by inactivating NLRP3 inflammasome, which was partially mediated through the SIRT3–SOD2–mtROS signaling pathway. In addition, Zebing Rao et al.^53^ have recently found that VD3 negatively regulates the NLRP3 inflammasome via vitamin D receptor (VDR) signaling that inhibits NLRP3 inflammasome activation by impeding its BRCC3‐mediated deubiquitination. Whether VD3 could attenuate NM‐induced NLPR3 inflammasome activation through the VDR signaling in keratinocytes remains to be explored.

**FIGURE 8:**
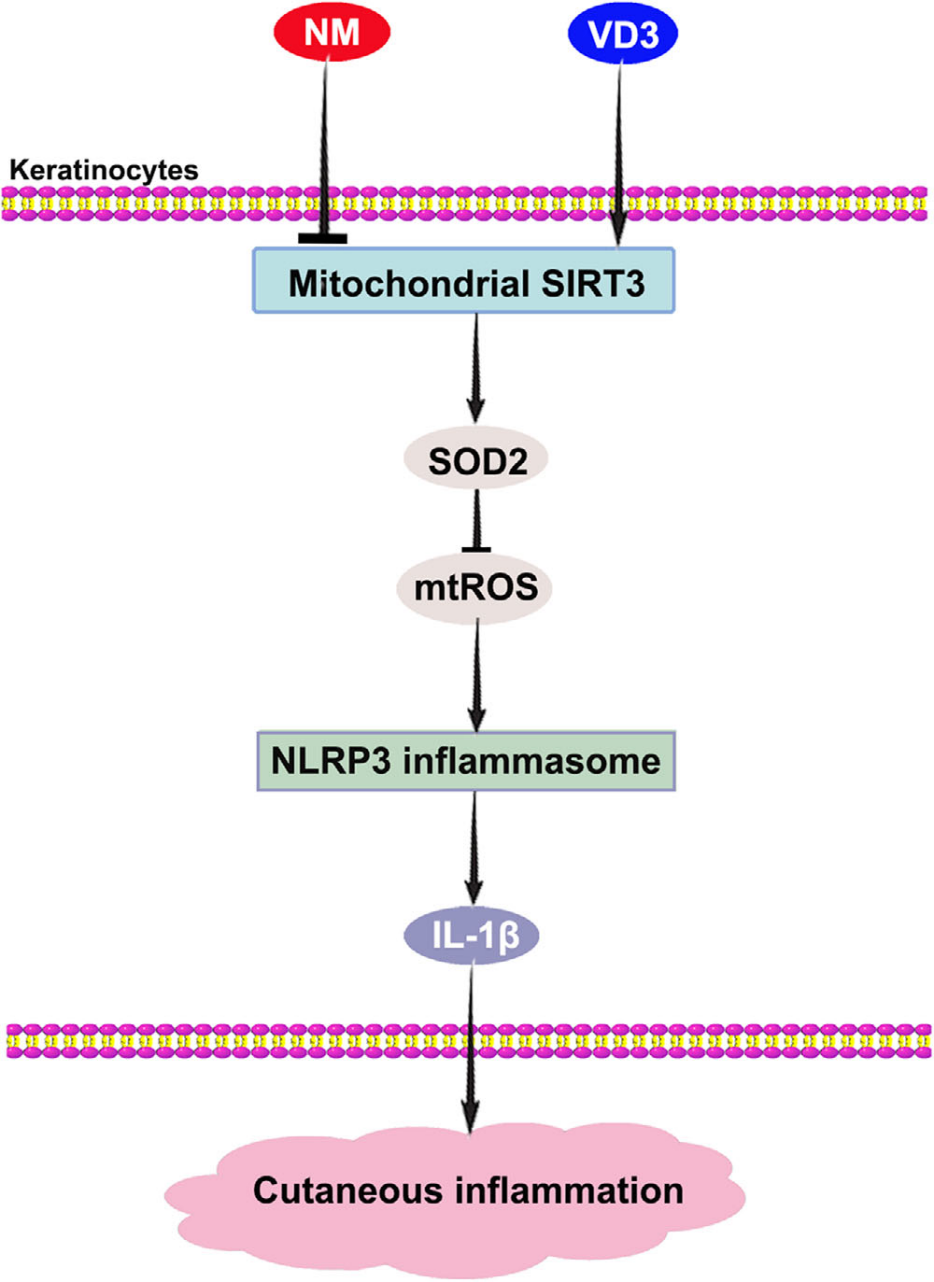
VD3 ameliorated NM‐induced cutaneous inflammation by inactivating the NLRP3 inflammasome through the SIRT3–SOD2–mtROS signaling pathway

Overall, we have uncovered a novel mechanism whereby NM induced cutaneous inflammation through mtROS‐dependent NLRP3 inflammasome activation. Interestingly, VD3 appears to exert beneficial effects on NM‐induced mtROS production and NLRP3 inflammasome activation through activating the SIRT3–SOD2 pathway (Figure 8). Our collective findings open a new avenue of research regarding the mechanism of VD3‐mediated protective effect on NM‐induced dermal toxicity, indicating that targeting SIRT3 or inhibiting NLRP3 inflammasome could be effective therapeutic strategies to combat vesicants‐induced skin injury.

## CONFLICT OF INTEREST

The authors declare no conflict of interest.

5

## Supporting information

Supporting information

## Data Availability

All data and materials are available to the researchers once published.
