# Supplementary material for: Vitamin D3 ameliorates nitrogen mustard‐induced cutaneous inflammation by inactivating the NLRP3 inflammasome through the SIRT3–SOD2–mtROS signaling pathway
Source: Clin Transl Med. 2021 Feb 14;11(2):e312. doi: 10.1002/ctm2.312 (PMC7882108; doi:10.1002/ctm2.312)
Supplement: Supplementary file 1 — Supporting information [file CTM2-11-e312-s001.doc]

**Supplementary Materials for:**

**Vitamin D3 ameliorates nitrogen mustard-induced cutaneous inflammation by inactivating the NLRP3 inflammasome through the SIRT3-SOD2–mtROS signaling pathway**

Xunhu Dong^1, 2^*, Ying He^4*^, Feng Ye^1,2^, Yuanpeng Zhao^1,2^, Jin Cheng^1,2^, Jingsong, Xiao^1,2^, Wenpai, Yu^1,2^, Jiqing Zhao^1,2^, Yan Sai^1,2^, Guorong Dan^1,2#^, Mingliang Chen^1,2,3#^, Zhongmin Zou^1,2#^

*These authors contribute equally to this work

^1^Department of Chemical Defense Medicine, School of Military Preventive Medicine, Third Military Medical University (Army Medical University), 30 Gaotanyan Street, Shapingba District, Chongqing 400038, China.

^2^Institute of Toxicology, School of Military Preventive Medicine, Third Military Medical University (Army Medical University), 30 Gaotanyan Street, Shapingba District, Chongqing 400038, China.

^3^Institute of Pathology and Southwest Cancer Centre, Southwest Hospital, Third Military Medical University (Army Medical University), Chongqing 400038, China.

^4^Department of Ultrasound, Xinqiao Hospital, Third Military Medical University (Army Medical University), Chongqing 400037, China.

**#Correspondence**

Guorong Dan, M.D., Ph.D., Department of Chemical Defense Medicine, School of Military Preventive Medicine, Third Military Medical University (Army Medical University), 30 Gaotanyan Street, Shapingba District, Chongqing 400038, China.

TEL: +86 2368771532; E-mail: [liroy@163.com](mailto:liroy@163.com)

Mingliang Chen, M.D., Ph.D., Department of Chemical Defense Medicine, School of Military Preventive Medicine, Third Military Medical University (Army Medical University), 30 Gaotanyan Street, Shapingba District, Chongqing 400038, China.

TEL: +86 2368771532; E-mail: [chenmingliang1987@163.com](mailto:chenmingliang1987@163.com)

Zhongmin Zou, M.D., Ph.D., Professor, Director, Department of Chemical Defense Medicine, School of Military Preventive Medicine, Third Military Medical University (Army Medical University), 30 Gaotanyan Street, Shapingba District, Chongqing 400038, China.

TEL: 86-23-68771526; FAX: 86-23-68771526; E-mail: [zmzou@tmmu.edu.cn](mailto:zmzou@tmmu.edu.cn).

**1. Materials and Methods**

**1.1 Reagents and antibodies**

Roswell park memorial institute (RPMI)-1640 Medium (SH30809.01B) and fetal bovine serum ([SH30370.03](http://www.bioon.com.cn/reagent/show_product.asp?id=1237790)) were purchased from Hyclone Laboratories (Logan, UT, USA). Mechlorethamine hydrochloride (a type of NM, K900001X) was obtained from Dibo. DMSO (D2650) and TEMPO (SML0737) were purchased from Sigma-Aldrich (St. Louis, MO, USA), 3-TYP (S8628) from Selleck Chemicals (Shanghai, China), YVAD (HY-P1009), MCC950 (HY-12815), anakinra ([HY-108841](https://www.medchemexpress.cn/anakinra.html)) and VD3 (HY-76915) from Med Chem Express (New Jersey, USA), and CCK-8 (CK04) from Dojindo Laboratories (Kumamoto, Japan). Human IL-1β ELISA kit (PI305), mouse IL-1β ELISA kit (PI301), human IL-6 ELISA kit (PI330), human TNF-α ELISA kit (PT518), DCFH-DA (S0033), DHE (S0063), caspase-1 activity kit (C1101), SOD activity kit (S0103) and anti-SIRT3 atibody (AF5303) were obtained from Beyotime Institute of Biotechnology. MitoSOX^TM^ Red (M36008) and Lipofectamine™ 2000 transfection reagent (11668-019) was acquired from Invitrogen (Carlsbad, CA, USA). Antibodies were purchased as follows: anti-IL-1β (12703/12242) and anti-SIRT3 (5490) from Cell Signaling Technology (Danvers, MA, USA), anti-SOD2 (sc-137254) and COX2 (sc-376861) from Santa Cruz Biotechnology (SantaCruz, CA, USA) and anti-acetylated SOD2 (ac-SOD2, ab137037), anti-AIM2 (ab119791), anti-NLRC4 (ab201792) and anti-NLRP3 (ab263899) from Abcam (Cambridge, MA). Caspase-1 antibody (NBP1-45433) was obtained from NOVUS Biologicals (Colorado, USA) whereas antibody against ACTB (TA-09) was obtained from Zhongshan Jinqiao Biotechnology Co (Beijing, China) and the SIRT3 assay kit (BPS-50088) from BPS Bioscience (San Diego, CA).

**1.2 Cell viability measurements**

HaCaT cells were purchased from Chinese Academy of Sciences Shanghai Cell Bank (cs0014, Shanghai, China) and cultured in RPMI-1640 supplemented with 10% fetal bovine serum in a humidified atmosphere containing 5% CO_2_ at 37℃. The medium was changed at 2-day intervals and cells re-plated at 80-90% confluence. The CCK-8 was employed to measure cell viability following the manufacturer’s protocol . Briefly, HaCaT cells in logarithmic growth period were seeded in a 96-well microplate (Corning Life Science, Corning, NY, 3650) at a density of 1×10^4^ cells per well. The medium was changed 24 h later, then cells were treated with NM at a series of concentrations (0, 1, 5, 10, 20, 50, 100, and 200 μM) for 4 h. Subsequently, CCK-8 solution (20 μL/well) was added to wells for 1.5 h. Viable cells were counted via absorbance measurements with a monochromator microplate reader (Safire II; Tecan Group Ltd., Männedorf, Switzerland) at a wavelength of 450 nm. The optical density value at 450 nm was determined as the percentage of cell viability in relation to the control group (set as 100%).

**1.3 Western blot analysis**

Cells and skin tissues were collected, lysed or homogenized for protein extraction and subjected to western blot, as described previously [^1^](#_ENREF_1). Briefly, 40-100 µg protein was resolved via 12-15% SDS-PAGE and electroblotted onto polyvinylidene difluoride membranes. Next, membranes were blocked with 5% skimmed milk and incubated overnight at 4 ℃ with primary antibodies at the following dilutions: SIRT3 (1:1000), SOD2 (1:1000), Ac-SOD2 (1:500), NLRP3 (1:1000), AIM2 (1:500), NLRC4 (1:800), caspase-1 (1:400), IL-1β (1:500), COX2 (1:500) and ACTB (1:1000). After washing three times with Tris-buffered saline containing 0.1% Tween 20 (TBST), membranes were incubated with the appropriate horseradish peroxidase-conjugated secondary antibodies ([Thermo Scientific Lab Vision;](http://www.thermoscientific.com/ecomm/servlet/search?searchType=0&searchSubType=6&N=4294967135 4294967089&Ne=4294967089&keyWord=rabbit+secondary+antibodies) 31340 and 31455). Following TBST washes, protein bands were visualized with electrogenerated chemiluminescence using the Vilber Fusion FX7 system.

**1.4 Measurement of the cellular IL-1β content**

After various treatments, IL-1β contents in culture supernatant fractions were determined with the human IL-1β ELISA kit following the manufacturer’s instructions. Absorption at 405 nm was assessed using an Infinite™ M200 Microplate Reader (Tecan Group Ltd.). Results were calculated based on a calibration curve generated with different concentrations of IL-1β standards.

**1.5 Measurements of enzymatic activity**

Cells in the logarithmic growth phase were seeded in six-well microplates, with three replicate wells for each condition. Cells subjected to the appropriate treatments were collected and enzymatic activities of SOD2, SIRT3 and caspase-1 were determined with commercial cell lysate assay kits, in keeping with the manufacturer’s instructions. Protein concentrations were assessed with a BCA protein assay kit (Beyotime, China). Absorption at 450 nm or 405 nm was measured using an Infinite™ M200 Microplate Reader (Tecan Group Ltd.).

**1.6 Small interference RNA assay**

HaCaT cells were transfected with *SIRT3* small interference RNA (siRNA, sc-61555), *NLRP3* siRNA (sc-45469), *caspase-1* siRNA (sc-29235) or negative control siRNA (sc-44230) for 5 to 7 h, in keeping with the manufacturer’s protocol. Cells were subsequently washed and incubated with fresh RPMI-1640 for a further 24 h. Next, cells were harvested for evaluation of target protein expression or incubated with specific reagents. At the end of the incubation period, cells were harvested and subjected to western blot and other analyses.

**1.7 ROS assessments in kerationocytes**

ROS or mtROS levels were measured using DCFH-DA (Beyotime) or MitoSOX^TM^ Red (Invitrogen) following the manufacturer’s instructions as described before [^1^](#_ENREF_1). Briefly, HaCaT cells were seeded in a 96-well microplate at a concentration of 10,000 cells/well and subjected to the indicated treatments. Cell-containing wells were loaded with DCFH-DA (10 μM) or MitoSOX reagent (5 μM) at 37℃ for 10 min in the dark, followed by three gentle washes in warm phosphate-buffer saline. Fluorescence intensities of total ROS and mtROS were determined using an Infinite^TM^ M200 Microplate Reader (Tecan Group Ltd.). Microscopic analyses were performed using a 440 Radiance 2000 laser scanning confocal microscope (Bio-Rad, Hercules, CA)

**1.8 H&E staining and histopathological analysis**

At 12 d after NM exposure, skin wounds and nearby tissues were removed and fixed in 10% formalin for histological analysis. Subsequent preparation for paraffin embedding was performed based on routine protocols. Slice thickness was limited to 5 μm for H&E staining and microscopic evaluation (Carl Zeiss, Germany) of histopathological features, such as epidermal thickness, parakeratosis, epidermal denuding and epidermal death, as described previously [^3^](#_ENREF_3).

**1.9** **Immunofluorescence analysis**

Cells were seeded in 15 mm confocal dishes and fixed with 4% paraformaldehyde for 10 min at room temperature. At 3 d after NM exposure, the wounded skin and nearby control tissues were excised in full-thickness and embedded in OCT at -20°C. Then, the tissues were sectioned at a thickness of 6-8 μm. For immunofluorescence analysis, the cells and sections were washed three times with phosphate-buffered saline for 5 min, permeabilized with 0.3% (v/v) Triton X-100 for 10 min and blocked in 3% BSA for 1 h at room temperature. Thereafter, the sections were incubated with rabbit anti-SIRT3 antibody (1:200) overnight at 4°C. Then, the sections were incubated with the appropriate Alexa Fluor® 488 goat anti-rabbit IgG (H+L) antibody (A11034, Invitrogen) for 2 h at room temperature. DAPI staining solution (C1005, Beyotime) was used to staining the nuclei for 10 min at room temperature. Finally, the sections were mounted on glass slide. All the digital images of the dishes and sections were acquired using a ZEISS LSM800 confocal laser scanning microscope (ZEISS, Germany).

**1.10 ROS assessments in skin**

To measure in situ ROS level, frozen sections (10 μm) of skin were stained with 5 μM DHE in PBS for 30 min, rinsed, mounted, and observed using a ZEISS LSM800 confocal laser scanning microscope (ZEISS, Germany), according to the previously validated method ^4^.

**1.11 Statistical analyses**

Quantitative data are presented as means ± standard deviation (SD) of three independent experiments. The statistical analysis was conducted with the *t*-test and one-way analysis of variance using SPSS 18.0 statistical software (SPSS Inc., Chicago, IL, USA). A *P*-value < 0.05 was considered statistically significant and the Tukey-Kramer post-hoc test was applied if *P* < 0.05.


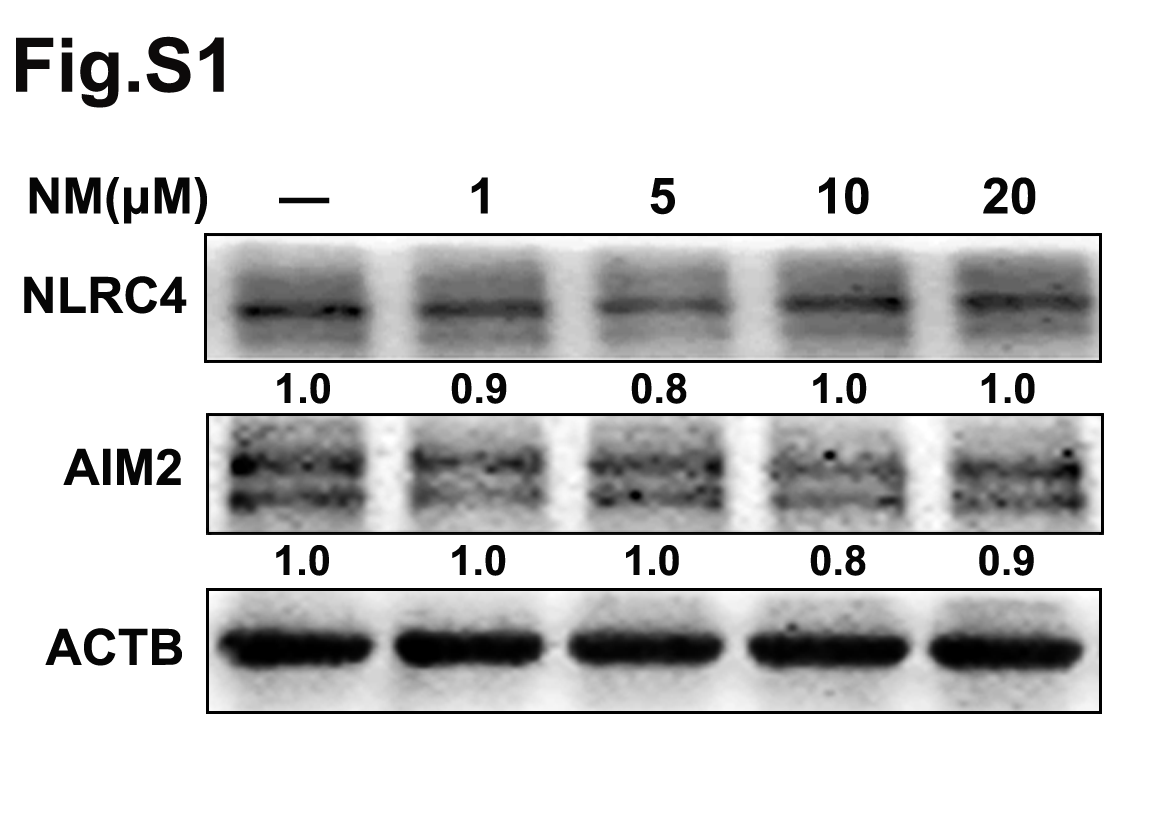


**Figure S1. The effect of NM on the expression of AIM2 and NLRC4, Related to Fig. 2.** HaCaT cells were treated with different concentrations of NM (0, 1, 5, 10, and 20 μM) for 4 h. NLRC4 and AIM2 expression were determined via western blot. Western blot results were quantiﬁed by densitometric analysis of representative immunoblots for NLRC4 and AIM2.


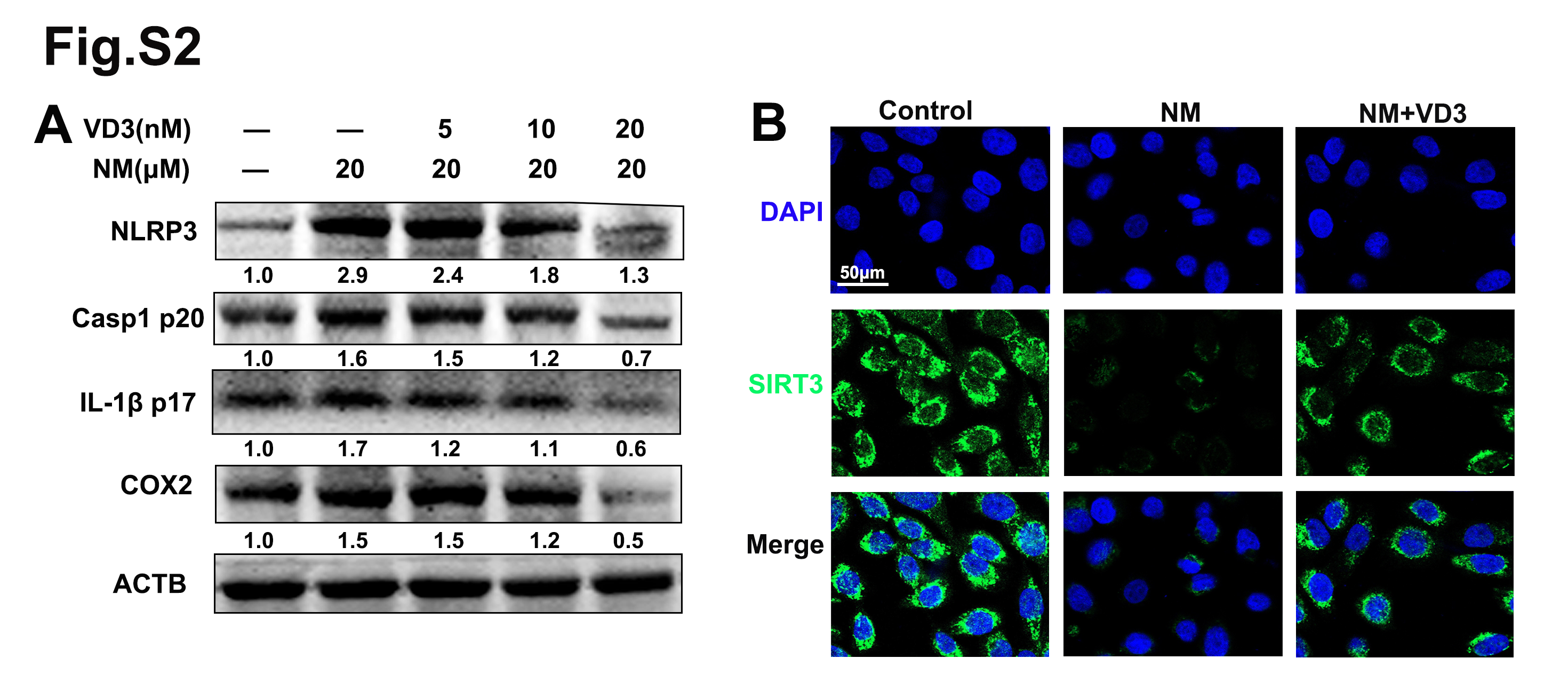


**Figure S2. The effect of VD3 on NM-induced NLRP3 inflammasome activation and SIRT3 inhibition, related to Fig. 5 and Fig. 6.** HaCaT cells were pre-treated with VD3 (0, 5, 10 and 20 nM) for 1 h, followed by treatment with NM (20 μM) for another 4 h. **(A)** NLRP3, Casp1 p20, IL-1β p17 and COX2 contents were determined by western blot and the representative immunoblots were quantiﬁed by densitometric analysis. **(B)** Immunofluorescence staining analysis of SIRT3 expression, Nuclei were counterstained with DAPI (blue).


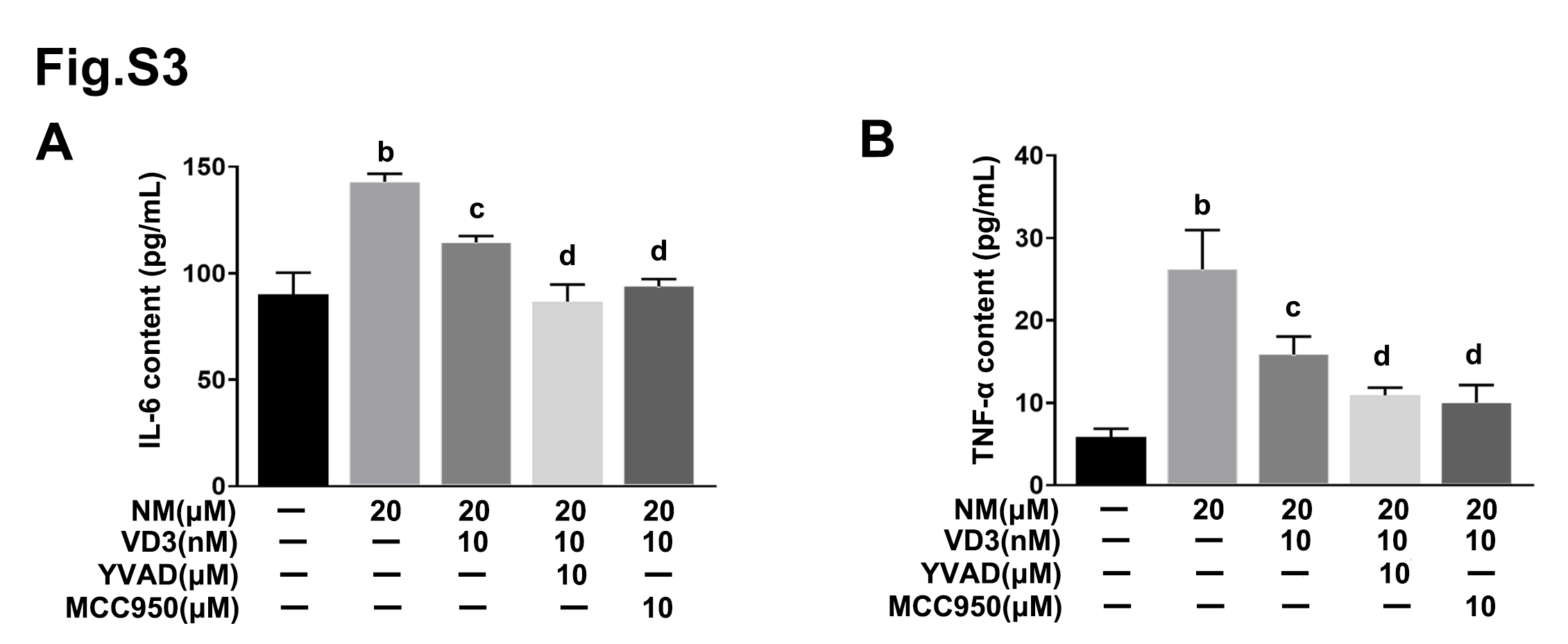


**Figure S3. The effect of YVAD and MCC950 on VD3-induced decrease of IL-6 and TNF-α content, Related to Fig. 5.** HaCaT cells were pre-treated with VD3 (10 nM) in the presence or absence of 3-TYP (10 μM) or MCC950 (10μM) for 1 h, followed by treatment with NM (20 μM) for another 4 h. ELISA measurement of **(A)** IL-6 and **(B)** TNF-α secretion in cell culture supernatant. Values are expressed as means ± SD (n = 3); ^b^*p* < 0.01 versus vehicle-treated control group (the black bar); ^c^*p* < 0.05 versus single NM-treated group; ^d^*p* < 0.05 versus NM and VD3 co-treated group.


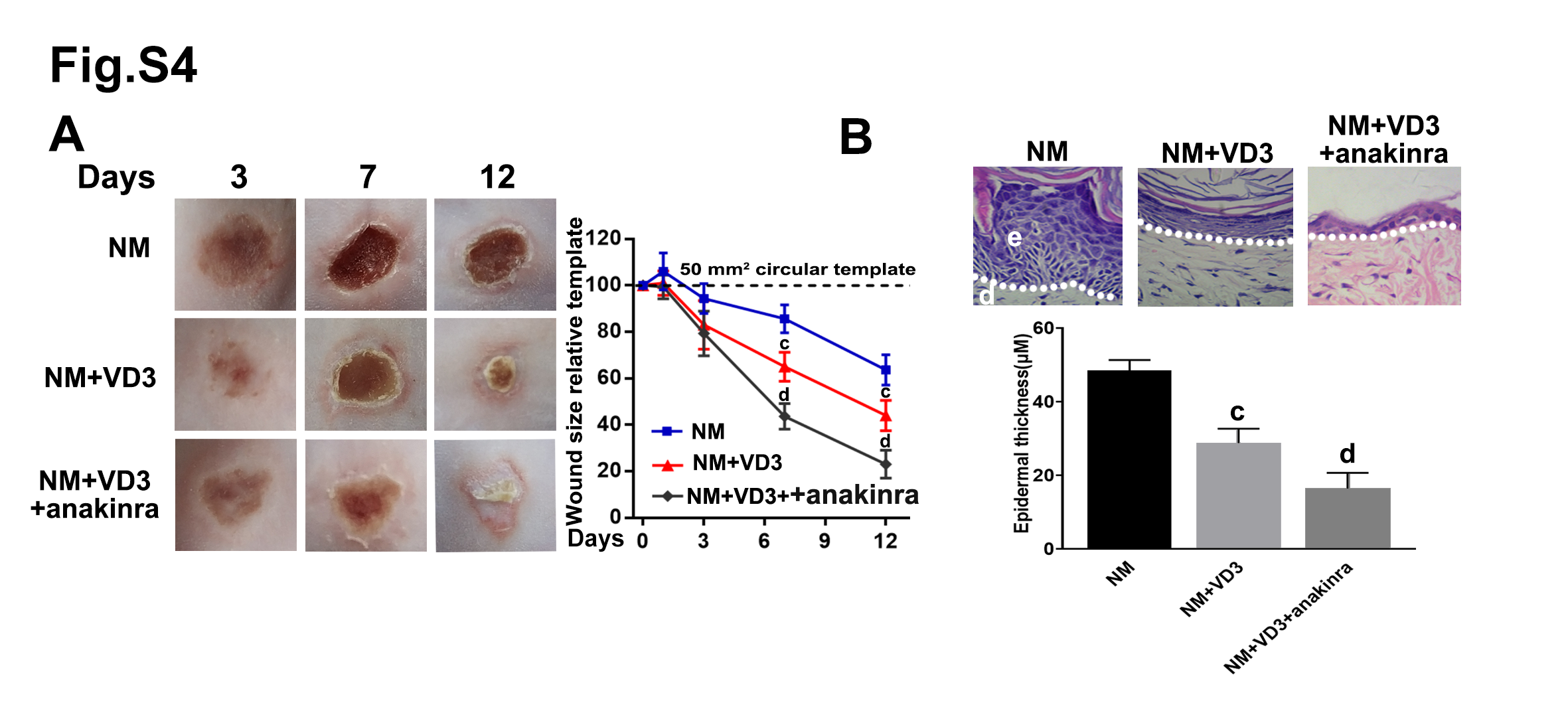


**Figure S4. The effect of anakinra on VD3-induced improvement of skin wound healing in NM-exposed mice, Related to Fig.7.** Dorsal skins of 8 week-old female C57BL/6J mice (n = 6 per group) were exposed to 3.2 mg NM in 200 μL acetone in the presence or absence of VD3 (50 ng /100 μL per mouse, i.p.) combined with or without anakinra (50 mg/kg) as described in Materials and methods. **(A)** Representative images of skin wound healing post-NM exposure. Mice were sacriﬁced at 12 d following NM exposure, and dorsal skin tissue was collected. **(B)** H&E staining was performed to analyze injured skin (400 × magnification). e, epidermis; d, dermis. ^c^*p* < 0.05 versus single NM-treated group; ^d^*p* < 0.05 versus NM and VD3 co-treated group.

**References**

1. Chen ML, Yi L, Jin X, et al. Resveratrol attenuates vascular endothelial inflammation by inducing autophagy through the cAMP signaling pathway. *Autophagy*. Dec 2013;9(12):2033-45. doi:10.4161/auto.26336

2. Chen ML, Zhu XH, Ran L, Lang HD, Yi L, Mi MT. Trimethylamine-N-Oxide Induces Vascular Inflammation by Activating the NLRP3 Inflammasome Through the SIRT3-SOD2-mtROS Signaling Pathway. *J Am Heart Assoc*. Sep 4 2017;6(9)doi:10.1161/JAHA.117.006347

3. Das LM, Binko AM, Traylor ZP, et al. Early indicators of survival following exposure to mustard gas: Protective role of 25(OH)D. *Toxicol Lett*. Apr 25 2016;248:9-15. doi:10.1016/j.toxlet.2016.02.013

4. Kim HR, Kim JH, Choi EJ, et al. Hyperoxygenation attenuated a murine model of atopic dermatitis through raising skin level of ROS. *PLoS One*. 2014;9(10):e109297. doi:10.1371/journal.pone.0109297
